# The cardiometabolic profile and related dietary intake of Ugandans living with HIV and AIDS

**DOI:** 10.3389/fnut.2022.976744

**Published:** 2022-08-11

**Authors:** Tonny Kiyimba, Fred Kigozi, Peter Yiga, Barbara Mukasa, Patrick Ogwok, Bart Van der Schueren, Christophe Matthys

**Affiliations:** ^1^Department of Food Technology, Faculty of Science, Kyambogo University, Kampala, Uganda; ^2^Clinical and Experimental Endocrinology, Department of Chronic Diseases and Metabolism, KU Leuven, Leuven, Belgium; ^3^Nutrition Unit, Department of Health Sciences, School of Applied Sciences, Mildmay Institute of Health Sciences, Kampala, Uganda; ^4^Mildmay Group, Mildmay Hospital, Kampala, Uganda; ^5^Department of Endocrinology, University Hospitals Leuven, Leuven, Belgium

**Keywords:** dietary intake, cardiometabolic, metabolic syndrome, HIV, AIDS, polyphenol

## Abstract

**Introduction:**

Suboptimal diet and physical inactivity downgrade the putative benefits of Antiretroviral Therapy (ART) among People Living with HIV (PLWH). However, there is paucity of literature on dietary intake and cardiometabolic profiles of PLWH in Uganda.

**Methods:**

A cross-sectional study among PLWH in Uganda was conducted. Dietary intake was assessed using a 24h recall method of 2 non-consecutive days. The short International Physical Activity Questionnaire assessed participants' physical activity. Fasted blood samples were analyzed for Fasting Blood Glucose (FBG), total cholesterol, LDL-c, HDL-c and triglycerides. Blood pressure and anthropometric measurements were performed following step 2 of the WHO STEPS.

**Results:**

253 patients completed in this study. A high prevalence of low HDL-c (31.9%), abdominal obesity (44.5%), high BMI (51.6%), raised FBG (45.3%), high SBP (31.5%), elevated triglycerides (26.4%) and metabolic syndrome (28%) was found. More women were identified with metabolic syndrome (31.5%) than men (19.2%). Low prevalence of high LDL-c (4.7%) and total cholesterol (9.8%) was found. Diets had a high carbohydrate (65.8 ± 10.4) E% and fiber intake (30.1 ± 12.7) g with minimal PUFA (6.1 ± 2.3) E%, fruits and vegetables (1.4 servings). High proportions were found of unmet intake for vitamin A (38.2%), B_1_(48.8%), B_2_ (29.6%), B_12_ (29%), folate (61.4%), Ca (76%), Zn (53.1%) and Mg (41.7%). Mean MET min was 6,700 ± 5,509 and over 68% of the participants had >3,000 MET min.

**Conclusion:**

Our findings reveal a high prevalence of metabolic disturbances among PLWH in Uganda and further highlight that their diets are suboptimal with low fruits and vegetable intake

## Introduction

By 2020, over 37.7 million people were living with HIV globally, with 67% of whom living in Sub Saharan Africa (SSA) (1). An estimated 1.4 million Ugandans are living with HIV and AIDS, with 38,000 new HIV infections recorded in 2020 while over 22,000 died of AIDS-related illnesses including non-communicable diseases (NCD) (2). Although the advent of Antiretroviral Therapy (ART) coincided with increased longevity and improvement in general quality of life of People Living with HIV (PLWH), sub-optimal cardiometabolic health among ART recipients are reported (3) and consequently increase the risk of age related NCD comorbidities (4). Moreover, the ART-associated cardiometabolic risks coupled with poor diet, physical inactivity, smoking and immoderate alcohol intake (5–8), now threaten to undo the earlier putative benefits of this therapy (9, 10). Subsequently, these factors can impose a cluster of negative metabolic changes collectively referred to as metabolic syndrome. Metabolic syndrome refers to a constellation of conditions that together increase the risk of heart disease, stroke and type 2 diabetes. Such metabolic conditions include high blood pressure, high blood sugar, excess body fat around the waist and dyslipidaemia. Metabolic syndrome heightens the risk of heart attack and stroke (11). A prevalence of metabolic syndrome of up to 21.5% has been noted in PLWH in sub-Saharan Africa (SSA) (12).

Although the cardio-metabolic profile of PLWH in Uganda has previously been reported to be suboptimal (13), the dietary intake of PLWH has not yet been adequately studied. Insights about the diet are a cornerstone in the optimisation of cardiometabolic health (14). However, presently there is a paucity of studies assessing cardiometabolic health and dietary intake of PLWH simultaneously. Therefore, this study aimed at characterizing the dietary intake and cardiometabolic profiles of PLWH in Community Drug Distribution Points (CDDPs) in Uganda.

## Materials and methods

### Study design and population

A cross-sectional study was conducted between May and July 2021 among PLWH in CDDPs in Wakiso district, central Uganda. Wakiso is one of the districts with the highest HIV and AIDS prevalence (7.3%) in Uganda (15). Study staff approached potential participants with verbal and written information about the study. Participants then provided signed informed consent in Luganda, the local language, or in English. In case of illiterate participants, a fingerprint was used to sign in the presence of a witness. Participants were requested to fast for at least 8 h and avoid strenuous activities on the day of measurement. The study protocol complied with the Helsinki declaration on human subjects (16) and was approved by Uganda National Council of Science and Technology (UNCST-HS1355ES).

#### Inclusion and exclusion criteria

Eligible participants were adults (≥18 years) living with HIV and AIDS, virally suppressed (HIV RNA <1,000 copies per mL of blood), not breastfeeding nor pregnant, with 95% ART adherence who had been on ART for at least 6 months in CDDPs. Patients co-infected with TB were excluded.

#### Outcome measures

Primary outcomes included dietary intake, and metabolic syndrome and its components (HDL-c, triglycerides, fasting blood glucose, waist circumference, body composition and blood pressure). Secondary outcome were BMI, total cholesterol and LDL-c.

#### Definitions

A reading ≥100 mg/dL was considered to be raised fasting plasma glucose, while ≥100 mg/dL to <126 mg/dL was categorized as prediabetes and ≥126 mg/dL was classified as diabetes (11). Low HDL-c was defined as <50 mg/dL in women or <40 mg/dL in men, readings ≥150 mg/dL were considered elevated triglycerides while blood pressure ≥130 ≥85 mm Hg was considered high (11). Cut points for fat mass were <25 kg for men or <35 kg in women (17) while waist circumference; <90 cm for men and <80 cm for women (18). Participants with BMI ranges (18.5–24.9 kg/m^2^) were considered normal, (≥25.0–29.9 kg/m^2^) overweight and (BMI ≥ 30 kg/m^2^) as living with obesity. Cut points for total cholesterol and LDL-c were <200 mg/dL and <140 mg/dL, respectively.

Metabolic syndrome was defined according to the NCEP/ATP III criteria (11), as existence of atleast three of the following CVD risk markers: raised fasting blood glucose (≥100 mg/dL); large waist circumference (>90 cm in men or >80 cm in women); high blood pressure (≥130 ≥85 mm Hg); elevated TG (≥150 mg/dL) and low HDL-C (<50 mg/dL in women or <40 mg/dL in men).

### Data collection

Data collection followed the WHO STEPS instrument for collecting data and measuring chronic disease risk factors (19). In step 1, a socio-demographic questionnaire was administered to elicit data on age, education level, tribe, marital status, occupation, socioeconomic status and household size. A specially developed self-reporting questionnaire was administered to collect medical information such as participants' date of HIV diagnosis, current combination of ART, duration on ART, prior diagnosis of metabolic disorders and current use of cholesterol stabilizing or performance enhancing drugs as well as the use of non-conventional medicines e.g., local herbs. Regarding ART regimen, a distinction was made between Protease Inhibitors (PI) or integrase inhibitor and non-Protease Inhibitors (non-PI) regimens. This information was later verified by reviewing patient's medical charts of each participant.

Physical activity was measured using the short form of the International Physical Activity Questionnaire (20). Participants that did not reach at least 600 metabolic equivalents of task (MET) were classified as physically inactive while a range ≥600 to <3,000 MET was considered minimally active. Individuals exceeding 3,000 MET were defined as having health-enhancing-physical activity. Alcohol intake was assessed using the WHO developed Alcohol Use Disorder Identification Test (AUDIT). A score of 8 or more is considered hazardous or harmful alcohol use with potential physical or physiological harm (21). Smoking frequency was measured based on previously validated questions on tobacco use (22, 23).

#### Dietary assessment and energy intake

Dietary intake data was collected by a non-consecutive 2 day 24-h dietary recall interview-based method by trained nutritionists. A 2 non-consecutive days method allows to assess an individual's usual intake (24). The interview allowed for estimation of food quantities and sizes and probing whenever required to ensure that foods were not forgotten. Participants were required to recall the specific timing of food consumption on each consumption day. During the interviews, time periods of consumption were structured as follows: breakfast, midmorning snack, lunch, evening snack, dinner, and night snack. Estimations of meal portion sizes were done by use of a photographic food atlas and household utensils (25). These proxy measurements were later converted into their equivalent metric units (grams) to quantify meal portion sizes. For determination of nutrient intake, the actual food intake was converted into relevant nutrients by use of Food Composition DataBases (FCDB). Due to the lack of a Ugandan FCDB, a combination of the Harvest Plus FCDB for central and eastern Uganda (26), the Kenyan and USDA FCBB (27) were used. The usual dietary intake was calculated using the Multiple Source Method (MSM) (28). In the absence of Dietary Recommended Intakes (DRIs) specific to patients with HIV and/or AIDS, data was compared to the general US Institute of Medicine nutrient recommendations (29, 30). Energy intake was compared with the Average Requirements (AR) of adults aged 19 and above. AR was a range of energy intakes based on a wide scope of Physical Activity Levels (PAL). The PALs used in this calculation were obtained from the physical activity assessment of the participants in this study. The dietary guidelines of the Therapeutic Lifestyle Changes of Nation Cholesterol Education Programme Adult Treatment Panel III (NCEP-ATP III-TLC) were used for recommendations of cholesterol, SFA, MUFA, soluble and insoluble dietary fibers (31). We used the FAO Global Individual Food Composition Data Tool to classify food into 14 different food groups (32). Total polyphenols intake was estimated using phenol explorer database (33).

#### Blood pressure and anthropometry

Height, weight, and blood pressure were measured according to step 2 of WHO STEPS. Waist circumference (to the nearest 0.5 cm) was measured using Gulick measuring tape at the level of the iliac crest with the participant standing, at the end of gentle expiration (34). Body composition was measured using Bodystat 1,500 lite touch.

#### Biochemical measurements

In WHO STEPS (step 3), fasting blood samples were collected and analyzed for glucose, total cholesterol, HDL-c, LDL-c and triglycerides using a CardioChek Plus. A laboratory technician drew a fingerstick blood sample of 15 to 40 μL onto the test strips.

### Statistical analysis

Considering the prevalence (21.5%) of metabolic syndrome among PLWH in SSA, a sample size calculation was performed, and 243 participants were required to give a statistical significance at 95% confidence interval. Data was analyzed by the Statistical Package for Social Science (SPSS) version 22 (IBM Corp, Armonk, NY, USA). We used the Shapiro-Wilk test to assess the normality of data. Possible associations between categorical data were analyzed using Pearson's Chi Square test. Gender differences in nutrient intake were determined by the student's *t*-test. Multiple logistic regression was used to determine the association between independent variables (ART duration and time lived with HIV energy and fiber intake) and metabolic syndrome and BMI at bivariate and multivariate levels. We could not assess the association between metabolic syndrome and ART regimen as 91% of the participants were on integrase inhibitor regimens. A *p*-value of < 0.05 was used for statistical significance.

## Results

Overall, out of the 273 participants recruited, 254 completed the study. In Table 1, demographic characteristics and the prevalence of modifiable CVD risk factors are presented. Majority (71.3%) of the participants were women.

**Table 1:** Sociodemographic characteristics and CVD modifiable risk factors of study population.

**Characteristics**	**Total (*N* = 254)**	**Male (*n* = 73)**	**Female (*n* = 181)**	***p-*value**
**Age (years), mean (SD)**				
Age	41.7 ±10.7	44.6 ± 10.8	40.4 ± 10.4	**0.004**
Age at time of diagnosis	32 ±10.1	35.4 ± 10.5	30.6 ± 9.6	**0.001**
**Household size (** ***n*** **, %)**				0.052
Living alone	24 (9.4)	11 (15.1)	13 (7.2)	
Multi-person households	230 (90.6)	62 (84.9)	168 (92.8)	
**Employment status (** ***n*** **, %)**				**0.017**
Employed	211 (83.1)	68 (98.3)	143 (79)	
Non-employed	43 (16.9)	5 (6.8)	38 (21)	
**Education level (** ***n*** **, %)**				0.598
None	79 (31.1)	24 (32.9)	55 (30.4)	
Primary school	89 (35)	21 (28.8)	68 (37.6)	
Secondary school	59 (23.2)	20 (27.4)	39 (21.5)	
Tertiary	27 (10.6)	8 (11)	19 (10.5)	
**Alcohol consumption**				**0.001**
Drinkers, *n*, (%)	85 (33.5)	38 (53.5)	47 (26)	
Non-drinkers, *n* (%)	169 (66.5)	35 (49.3)	134 (74)	
Heavy drinkers (AUDIT score ≥8), *n*, (%)	36 (14.2)	8 (11)	28 (15)	0.351
**Smoking**				**0.001**
Non-smokers, *n*, (%)	240 (94.5)	63 (86.3)	177 (97.8)	
Smokers, *n*, (%)	14 (5.5)	10 (13.7)	4 (2.2)	
**Physical activity**				
MET (Mean, SD)	6,700 ±5,509	7,567 ±5,534	6,350 ±5,475	0.111
Inactive (<600), (*n*, %)	31 (12.2)	6 (8.2)	25 (13.8)	
Minimally active (600 <3,000), (*n*, %)	51 (20.1)	9 (12.3)	42 (23.2)	
HEPA active (≥3,000), (*n*, %)	172 (67)	58 (79.5)	114 (63)	

*SD, Standard Deviation; AUDIT, Alcohol Use Disorder Identification Tool; MET, Metabolic Equivalent of Task; HEPA, Health Enhancing Physical Activity; Significant differences (p < 0.05) are represented with values in bold*.

The time lived with HIV and AIDS among participants was on average 9.6 ± 7.3 years and participants had been on ART for an average duration of 8.5 ± 6.3 years. Of all participants, 91% reported to be taking Dolutegravir/Lamivudine/Tenofovir disoproxil (DTG/3TC/TDF) class of ART while 5.1% were being treated with Tenofovir disoproxil fumarate/Lamivudine/Efavirenz (TDF/3TC/EFV) ART combinations. Use of complementary and alternative medicine was reported in 40.6% of the participants. These included local herbs e.g., *Aloe barbadensis miller, Hibiscus sabdariffa, Cassia obtusifolia* and *Tamarindus indica*.

Over 33% of the participants consumed alcohol and the consumption ranged from 1 to 10 bottles of beer on a single drinking occasion. On average females had a higher AUDIT score than men. Cigarettes were the only form of tobacco use reported. The average number of cigarettes smoked per day was 7 and ranged from 1 to 20 cigarettes while the average number of cigarettes smoked on heaviest smoking days was 10. On average, participants began smoking at 22 years of age and had been smoking for 21 years. The MET min ranged from 150 to 22,932 with only 12.2% below the 600 MET threshold.

**Figure 1:**
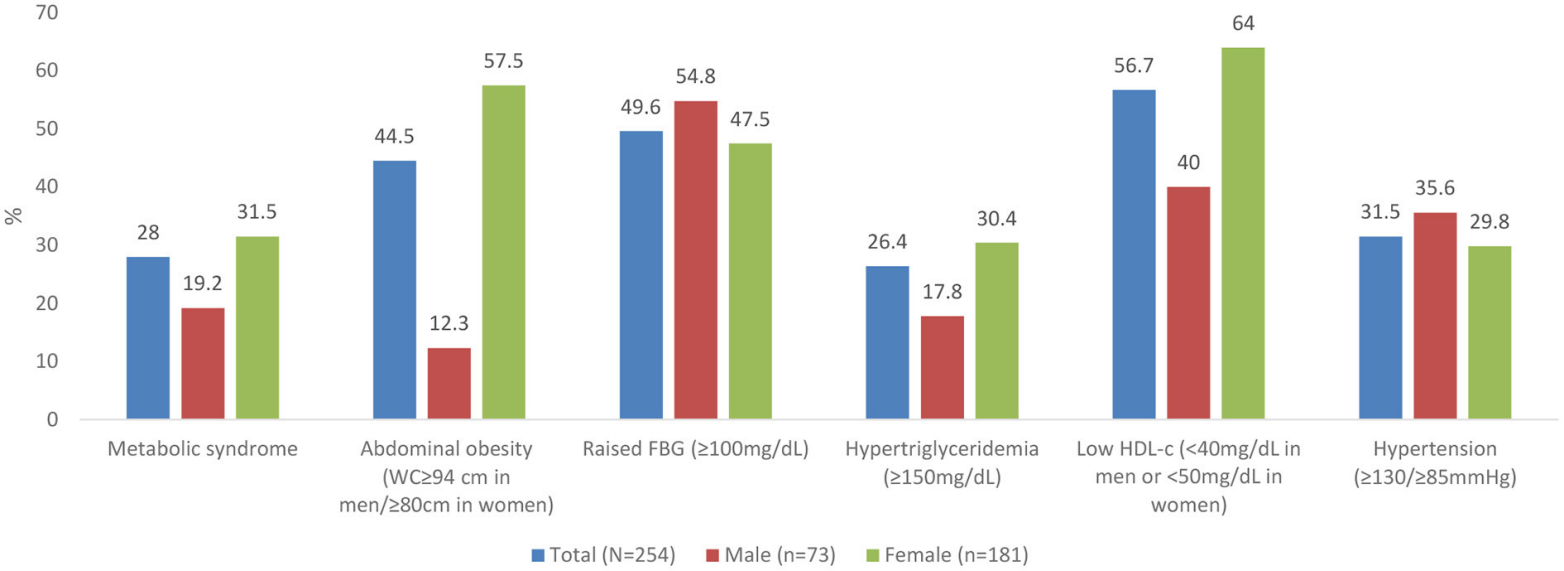
Prevalence of metabolic syndrome and its components. WC, waist circumference; FBG, Fasting Blood Glucose; HDL, High Density Lipoproteins.

The prevalence of metabolic syndrome and its components are presented in Table 2 and Figure 1. In total, 28% of the participants met the criteria for metabolic syndrome and this was significantly higher in women (31.5%) than in men (19.2%), (*p* = 0.048). The prevalence of abdominal obesity, raised FBG, and low HDL-c was, respectively, 44.5, 49.6, and 56.7%. Women had significantly higher prevalence of central obesity (p=0.035) and hypertriglyceridemia (*p* = 0.014), respectively, than men. Of the 49.6% (*n* = 126) participants with FBG > 100 mg/dL, 20.6% (*n* = 26) participants had FBG exceeding 126 mg/dL. Although results did not reach statistical significance, hypertension was more prevalent in men than women. Other additional cardiometabolic risk factors; fat mass, BMI, LDL-c and total cholesterol are summarized in Table 2. Overall, all participants had low total cholesterol and LDL-c levels with <10% of the participants exceeding the upper reference value for both. More than half of the participants had a BMI higher than 24.9 kg/m^2^. Of these, 21.2% (*n* = 54) had a BMI higher than 29.9 kg/m^2^. Proportions of metabolic syndrome and its components and high BMI stratified by age, are shown in Supplementary Table 1. In terms of fat mass, the proportion of men exceeding the reference value was higher than women (50.7 vs. 12.2%, *p* = 0.032). In multivariate analysis, there was no significant association between duration of ART, years lived with HIV, energy or fiber intake with metabolic syndrome or a high BMI.

**Table 2:** Mean cardiometabolic profiles by sex.

**Cardio-metabolic profile characteristics**	**Total (*****n*** = **254)**		**Male (*****n*** = **73)**		**Female (*****n*** = **181)**		***P*-value**	**Reference value (RV)**	
	**mean** **(SD)**	**%** **Exceeding** **RV**	**mean** **(SD)**	**%** **Exceeding** **RV**	**mean** **(SD)**	**%** **Exceeding** **RV**		**Men**	**Women**
**Anthropometry**									
Waist circumference (cm)	82.8 (11.6)	44.5	80.6 (9.6)	12.3	83.7 (12.1)	57.5	0.035	<94	<80
BMI (kg/m^2^)	26.2 (5.3)	51.6	26.7 (5.4)	58.9	26.1 (5.3)	48.6	0.421	18.5–24.9	
**Body composition** **(%)**									
Fat Mass	24.1 (10.0)	23.2	26.2 (10.5)	50.7	23.2 (9.6)	12.2	0.032	<25	<35
**Blood sugar profile** **(mg/dL)**									
FBG	105.8 (38.4)	45.3	102.7 (22.4)	54.8	107.1 (43.4)	47.5	0.411	<100	
**Blood lipid profile** **(mg/dL)**									
Total cholesterol	154.1 (38.4)	9.8	137.2 (36.8)	6.8	160.9 (37.1)	38.2	0.001	<200	
Triglycerides	131.6 (78.6)	26.4	112.6 (61.7)	17.8	139.3 (83.4)	30.4	0.014	<150	
HDL-c	47.7 (12.4)	31.9	45.5 (13.1)	38.4	48.6 (12)	16.6	0.071	>40	>50
LDL-c	84.6 (32.4)	4.7	57.6 (42.8)	4.1	80.6 (38.9)	6.1	0.001	<140	
Non-HDL-c	111.1 (34.7)	7	100.8 (34.7)	5.5	114.5 (34.1)	7.7	0.009	<160	
LDL:HDL	1.9 (1.3)	2.8	1.7 (0.8)	15.1	1.9 (1.4)	42.5	0.172	<2.5	<2
Tc:HDL	3.4 (0.9)	83.1	2.5 (1.5)	23.3	3.3 (1.1)	64.6	0.001	<3.0	<3.5
**Blood pressure** **(mmHg)**									
SBP	122.9 (12.1)	31.5	123.9 (11.4)	35.6	122.5 (12.4)	29.8	0.426	<130	
DPB	74.5 (7.4)	5.5	73.8 (7.1)	4.1	74.8 (7.5)	20.4	0.341	<85	

*Reference values are according to NCEP-ATP III and IDF; BMI, Body Mass Index; FBG, Fasting Blood Glucose; HDL-c, High Density Lipoprotein cholesterol; LDL-c, High Density Lipoprotein cholesterol; Tc, Total cholesterol; SBP, Systolic Blood Pressure; DBP, Diastolic Blood Pressure; SD, Standard Deviation; Significance at 0.05 level*.

**Figure 2:**
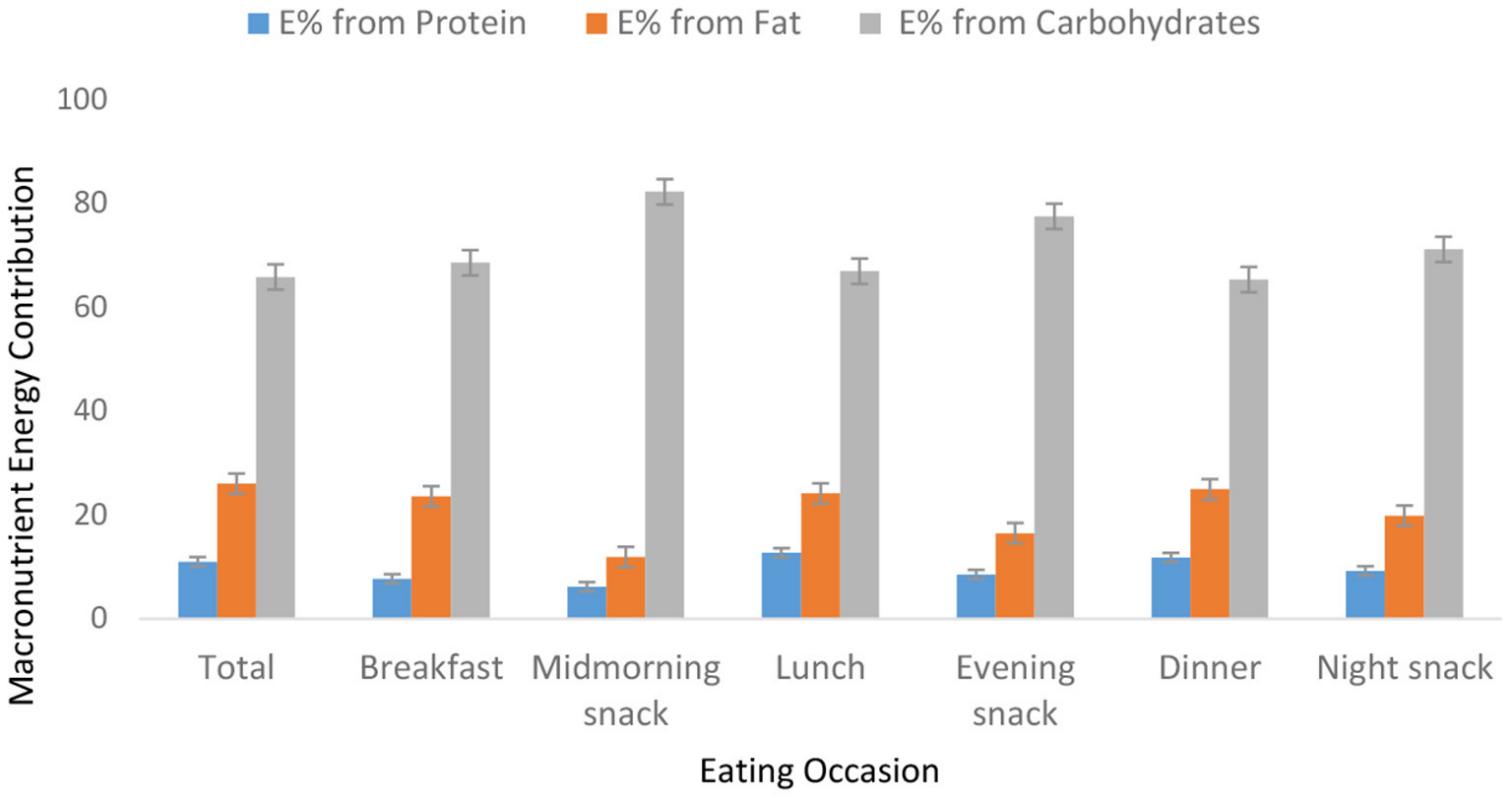
Energy contribution per macronutrient per eating meal.

**Figure 3:**
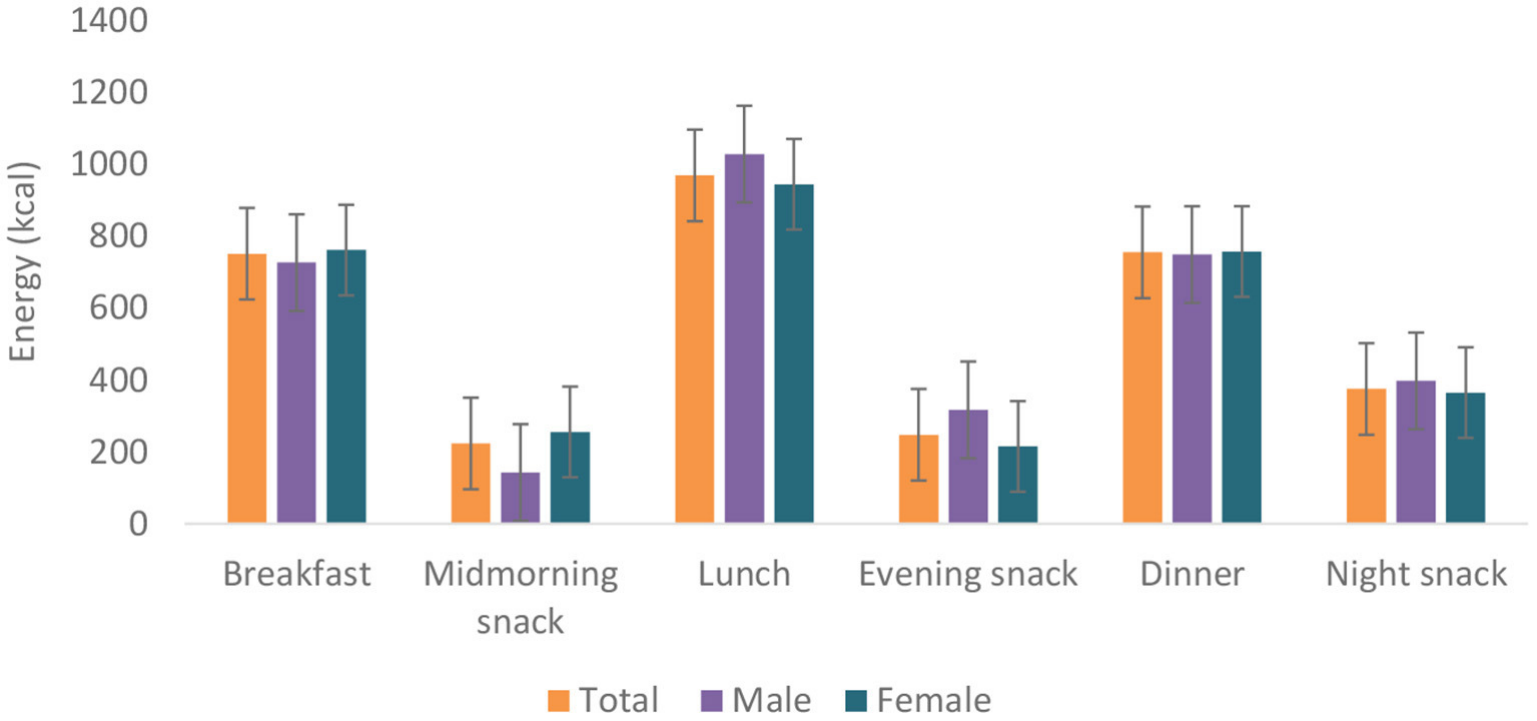
Energy distribution per eating occasion.

Food group consumption is presented in Table 3 and Supplementary Figure 1. Mean daily consumption was highest for roots, tubers, and plantains (528 g), followed by 239.4 and 186.5 g for cereals and legumes, respectively. The daily intake of fruits and vegetables was reported to be a serving or less. The mean daily energy intake was 2,389 (±768) kcal, and this came from breakfast (751 ± 459 kcal), lunch (969 ± 516 kcal) and dinner (755 ± 551 kcal). Over 19% of the participants had daily energy intake exceeding their Average Requirement. Energy and macronutrient intake for both sexes compared with the dietary recommendations are summarized in Table 4. There was a significant difference in protein intake across sex (men 72.6 vs. women 59%, *p* = 0.031). Our results show that in total, 76.8% of the participants had fat intake above 20 E%. A PUFA intake below the IOM recommendation of 5–10 E% was found in 36.6% of the participants. Most participants (80.7%) had a cholesterol intake of lower than 200 mg. There were significant differences (*p* = 0.029) in cholesterol intake across sexes (men 152.5 vs. women 122.8 mg). The energy and total dietary fiber distribution per eating occasion are shown in and Supplementary Table 2. Regarding energy contribution by macronutrients across all eating occasions (Figure 2), carbohydrates contributed the highest proportion of energy. None of the eating occasions had a carbohydrate-derived energy contribution lower than 65 E% with the largest E-intake of carbohydrates taken during midmorning snack (82 E%). Overall, lunch contributed the highest amount of energy of all the eating occasions (Figure 3).

**Table 3:** Usual intake of different food groups by sex.

**Food groups (g)**	**Total (*****N*** = **254)**		**Men (*****n*** = **73)**		**Women (*****n*** = **181)**		***P*-value**
	**Mean**	**SD**	**Mean**	**SD**	**Mean**	**SD**	
Cereals and their products	239	201	269	209	227	197	0.136
Roots, tubers, plantains and their products	528	350	492	264	543	379	0.303
Pulses, seeds and nuts and their products	187	101	114	120	216	120	0.469
Milk and milk products	147	236	156	275	143	219	0.681
Eggs and their products	19	46	19	43	19	48	0.997
Fish, shellfish and their products	88	126	78	131	92	125	0.426
Meat and meat products	121	144	141	152	113	140	0.013
Insects, grubs and their products	0	0	0	0	0	0	
Vegetables and their products	111	98	117	111	108	92	0.428
Fruits and their products	85	143	82	139	86	145	0.814
Fats and oils	19	23	19	27	19	21	0.922
Sweets and sugars	62	58	54	58	65	58	0.161
Spices and condiments	16	115	28	198	11	51	0.286
Beverages	757	433	723	438	771	432	0.432

*SD, Standard Deviation; Significance at 0.05 level. The food groups were defined according to the FAO/WHO| Global Individual Food consumption data Tool (32)*.

**Table 4:** Usual energy and macronutrients intake by sex and comparison with the IOM recommendations.

	**Total (*****N*** = **254)**		**Men (*****n*** = **73)**		**Women (*****n*** = **181)**		***P*-value**	**Dietary Recommendations (DR)**	
	**Mean (SD)**	**% Achieving DR**	**Mean (SD)**	**% Achieving DR**	**Mean (SD)**	**% Achieving DR**		**Men**	**Women**
Energy intake (kcal)	2,389 (±768)	49.2	2,469 (±676)	42.5	2,356 (±802)	51.9	0.292	1,786–2,947	1,611–2,942
**Macronutrients**									
Water (l)	1.5 (±0.5)	0.8	1.5 (±0.5)	0	1.5 (±0.5)	1.1	0.705	3.7 L*	2.7 L*
Protein (E%)	11 (±2.5)	63	11.5 (±2.7)	72.6	10.8 (±2.4)	59	0.031	10–35 E%	
Total Carbohydrates (E%)	65.8 (10.4)	96.1	64.6 (±11.1)	95.9	66.3 (±10.1)	96.1	0.231	45–65 E%	
**Dietary fiber (g)**									
19–50 years	30.1 (±12.7)	42.9	29.6 (±11.8)	15.4	29.6 (±11.5)	63.2	0.893	38 g*	25 g*
>51 years			31 (±11.7)	38.1	33.6 (±19.6)	79.3		30 g*	21 g*
Soluble dietary fiber (g)	9.4 (±3.4)	90.2	10.1 (±3.5)	78	9.7 (±3.4)	95	0.465	7.5	5.25
Insoluble dietary fiber (g)	21 (±9.8)	57.9	20.5 (±9)	31.5	21.2 (±10.2)	68.5	0.623	22.5	15.75
Fat, total (E%)	26 (±7.9)	76.8	26.2 (±8.3)	74	25.9 (±7.7)	77.6	0.755	20–35 E%	
SFA (E%)	7.1 (±2.9)	53.1	7.6 (±3.3)	50.7	6.9 (±2.7)	54.1	0.102	<7 E%	
MUFA (E%)	6.8 (±2.4)	100	7.2 (±2.3)	100	6.7 (±2.4)	100	0.137	<20 E%	
PUFA (E%)	6.1 (±2.3)	63.4	6 (±2.4)	60.3	6.1 (2.3)	64.6	0.879	5–10 E%	
Cholesterol (mg)	131.3 (±98.2)	80.7	152.5 (±109.1)	71.2	122.8 (±92.4)	84.5	0.029	<200 mg	

The reference for dietary recommendations (energy intake, water, protein, carbohydrates, fat, total dietary fiber, total fat and PUFA) is the Institute of Medicine. The average requirement of energy intake for both men (length 150–178 cm, aged 19 to>70) and women (length 140–175 cm, aged 19 to >70) is a range of energy intakes based on PAL 1.4–2.4. SFA, saturated fatty acids; MUFA, mono-unsaturated fatty acids; PUFA, poly-unsaturated fatty acids; SD, standard deviation; (*) Adequate intake. The DR for Cholesterol, SFA, MUFA, soluble and insoluble dietary fibers are according to NCEP-ATP III-TLC (30).

Ca, Mg, Zn and vitamins A, B_1_, B_2_ and folate had an intake below the recommendations (Table 5). None of the participants had an intake below the EAR or AI for sodium or potassium. Inadequate iron intake was found in over 37% of women.

**Table 5:** Usual micronutrient intake by sex and compared with IOM recommendations.

**Micronutrients**	**Total (*****N*** = **254)**		**Men (*****n*** = **73)**		**Women (*****n*** = **181)**		***P*-value**	**Dietary recommendations (DR)**	
	**Mean (SD)**	**% Not** **achieving DR**	**Mean (SD)**	**% Not** **achieving DR**	**Mean (SD)**	**% Not** **achieving DR**		**Men**	**Women**
**Minerals**									
Selenium (μg)	75.3 (22.6)	3.9	81.4 (±21.5)	4.1	72.9 (±22.6)	8.8	0.006	45	
Potassium (mg)	3,923 (±13,223)	0	4019.2 (±1340.6)	0	3884.8 (±1317.5)	0	0.465	3,400*	2,600*
Sodium (mg)	5,111 (±15,823)	0	5287.2 (±1585.3)	0	5039.7 (±1580.2)	0	0.260	1,500*	
Magnesium (mg)	331(±137.6)	41.7	357.1 (±136.2)	60.3	321 (±137.2)	34.3	0.063	350	255
Zinc (mg)	7.9 (±3.4)	53.1	8.9 (±3.4)	69.9	7.6 (±3.4)	46.4	0.006	9.4	6.8
Calcium (mg)	670.2 (±472)	76	660.9 (±484.8)	72.6	674 (±468.1)	77.3	0.842	800	
**Iron (mg)**									
19–50 years	10.7 (±3.6)	20	11.1 (±3.4)	6.8	10.3 (±3.2)	37.6	0.198	6.0	8.1
>51 years					11.7 (±5.6)	0		6.0	5.0
**Vitamins**									
Vitamin A (μg)	653 (±322.4)	38.2	652.2 (±399.9)	56.2	653.3 (±286.5)	30.9	0.981	625	500
Vitamin B12 (μg)	3.7 (±2.6)	29	3.9 (±2.5)	23.3	3.7 (±2.7)	30.9	0.569	2.0	
Folate (μg)	306.4 (±111.3)	61.4	311.1 (±115.2)	61.6	304.5 (110)	61.9	0.669	320	
**Vitamin B6 (mg)**									
19–50 years	2.1 (±0.7)	13	2.1 (±0.6)	5.8	2.0 (±0.62)	21	0.333	1.1	1.1
>51 years			2.2 (±0.8)	9.5	2.3 (±0.9)	7.1		1.4	1.3
Riboflavin (mg)	1.5 (±0.8)	29.6	1.5 (±0.8)	32.9	1.5 (±0.9)	27.6	0.480	1.1	0.9
Thiamine (mg)	1.0 (±0.3)	48.8	1.0 (±0.3)	49.3	0.9 (±0.3)	48.6	0.090	1.0	0.9
Niacin (mg)	15.1 (±4.7)	19.3	15.8 (±4.5)	20.5	14.9 (±4.7)	18.8	0.162	12	11
Vitamin C (mg)	108 (±40.9)	15	107.9 (±43.5)	24.7	108.1 (±39.9)	11	0.968	75	60

Reference intake is Estimated Average Requirement or Adequate Intake (*); SD, Standard Deviation.

Our results Table 6 show that the usual intake of total polyphenols was 212 (±283) mg, beans contributed the highest amount of total polyphenols followed by cereals, peanuts, vegetables and fruits.

**Table 6:** Usual total polyphenol intake (mg) and related food sources.

**Food items**	**Total**		**Men**		**Women**		***P*-value**
	**Mean**	**SD**	**Mean**	**SD**	**Mean**	**SD**	
Overall total polyphenol intake	212	283	230	295	194	275	0.112
Fruits and fruit products	133	214	155	241	110	201	0.157
Tea	7	7	6	6	7	7	0.311
Cooking oil	3	6	3	5	3	6	0.851
Spices and herbs	10	51	8	37	13	56	0.533
Peanuts	211	380	187	329	235	399	0.365
Beans	1,079	1,354	1,208	1,473	949	1,300	0.170
Vegetables and vegetable products	144	213	155	265	133	188	0.450
Roots and tubers	55	104	67	122	42	94	0.117
Beer	11	61	14	88	7	46	0.406
Cereals and cereal products	465	435	493	380	437	455	0.352

*Conversion factors for total polyphenols are derived from Phenol explorer version 3.1 (33)*.

## Discussion

The major findings were high prevalence of low HDL-c, abdominal obesity, raised FBG, hypertension and elevated triglycerides. These metabolic abnormalities ultimately culminated into a high prevalence of metabolic syndrome (28%). Significant sex differences were observed as more women identified with metabolic syndrome, abdominal obesity, low HDL-c and elevated triglycerides than men. Remarkably, majority of the study participants had optimal levels of LDL-c and total cholesterol. Generally, diets were characterized by a large intake of roots and tubers, whole cereals, legumes with minimal fruits and vegetables. As a result, diets were high in carbohydrate and fiber but deficient in several vitamins, minerals and PUFA.

Overall, half (51.6%) of the participants were either living with overweight or obesity while 44.4% had abdominal obesity. Women posted substantially higher rates of abdominal obesity than men. A similar trend has been reported in the general Ugandan population (35). There is strong evidence across SSA that obesity and abdominal obesity exist at high levels especially among women (13, 36–39). A lower prevalence of obesity has been reported in high income countries e.g., 38% in UK (40) and 20.4% in France (41). The cause for the observed overweight/obesity and abdominal obesity can be twofold. First, it could be due to the health-beauty paradox, a sociocultural misconception in SSA where a big body size has been misconstrued for wealth, beauty, respect and freedom from HIV (42–44). This paradox fosters unhealthy lifestyles and thwarts willingness to lose weight (45, 46). However, HIV and ART could equally contribute to obesity and abdominal obesity. ART reduces proinflammatory cytokines consequently resulting into mild to moderate insulin resistance. The decrease in insulin resistance in adipose tissue increases glucose uptake and lipid metabolism, a precursor for weight gain and visceral obesity (47). Different ART classes can confer negative metabolic response e.g., PIs, InSTI and NRTI are associated with weight gain and fat redistribution (48). DTG/3TC/TDF which is currently the most preferred first and second line of ART (49) has been found to increase body weight and BMI in even ART naïve PLWH (50). However, the weight gain under Dolutegravir as a monotherapy or when used as DTG/3TC/DTF combination is still lower than what is experienced in PIs and NNRTI based regimens (51).

Likewise, HIV etiology and ART have been shown to exacerbate metabolic syndrome. Although the pathophysiology remains rather elusive, HIV triggers glucose metabolism dysregulation and dyslipidaemia. In part, this is linked to the inflammation triggered by viral infection (52). The virus triggers chronic activation of the innate immune system with excessive production of inflammatory cytokines. These inflammatory mediators increase the risk of atherosclerosis (19) and insulin resistance (47). Additionally, ART affects cis-9-retinoic acid synthesis, resulting into dysregulation of adipocyte differentiation, apoptosis and increased hepatic triglyceride (53). The metabolic syndrome encountered in our study is higher than the 21.5% reported among PLWH in SSA (12). Metabolic syndrome arguably exists at high levels among PLWH in Uganda (5). Our findings on other components of metabolic syndrome are consistent with related studies investigating cardiometabolic risks among PLWH in Uganda (5, 13, 54). However, the reported prevalence of raised FBG is in stark contrast to findings from other SSA settings (5, 13, 37). This variation could stem from the differences in the analytical methods used, laboratory verses point of care testing (POCT), the latter often overestimates blood glucose (55). In addition, age, ART treatment regimen, duration on ART and time lived with HIV all of which have been shown to affect FBG differed considerably across these studies. High prevalence of low HDL-c among Ugandans appears to be a rather common occurrence. This is evidenced by the 2014 Uganda STEPS survey that reported a high prevalence (59.9% in men vs. 68.3% in women) of low HDL-c among the general Ugandan population (22). On the other hand, the observed low prevalence of LDL-c and total cholesterol may be linked to the high physical activity levels and fiber intake among our study participants. Both intensive and moderate exercises have been shown to significantly reduce total cholesterol and LDL-c in intervention studies (56). Likewise, dietary fibers particularly from whole grains have demonstrated beneficial cholesterol regulation functions (11). Noteworthy, the fiber intake in our study is generally higher than what is seen among Western populations (57).

Similar to other East African countries (58), diets in our study were predominantly carbohydrate-based and mainly roots, tubers, plantain and cereals and legumes. Intake of fruits and vegetables was four times lower than the WHO recommendation of at least 5 servings per day (59). The 2014 STEPS survey shows that consumption of fruits and vegetables among Ugandans is limited to just 1.4 servings a day with 88% of Ugandans not meeting the recommended fruits and vegetable intake while 27% do not eat fruit or vegetables a week (22). There is a sociocultural misconception toward fruit and vegetable intake among Ugandans where fruits are considered snacks for children and vegetables for poor people (42). In our study, women were at a higher risk of dietary protein inadequacy than men. The eating out of home tradition of men gives them access to foods of animal sources (60). The low intake of PUFA among our study participants could explain the high prevalence of low HDL-c observed (61).

Micronutrients have pivotal physiological functions in immune responses that influence HIV disease suppression consequently reducing viral load and overall mortality (60, 62). As such, quality of diet ameliorates micronutrient nutrition among PLWH (63). More than half of the participants had an intake below the EAR for Zn and Ca. On the other hand, adequate intake was met for vitamin C, K, Na and Se. Vitamin C and Se are potent antioxidants and enhance recovery of symptomatic PLWH (60). Specifically, vitamin C stimulates interferon production; a protein responsible for protecting cells against viral damage (60, 64). In regard to the B group vitamins, highest dietary deficiencies were observed for folate, B_1_, B_2_, B_12_ and B_3_. Adequate intake of B group vitamins is associated with improved clinical outcomes of HIV treatment and overall wellbeing of PLWH (65). In fact, vitamin B can reduce viral load and block the progression of HIV to AIDS (65–67). Inadequate dietary micronutrient intake especially vitamins A, C, riboflavin, folate and minerals Ca, Zn and Fe among PLWH is widely reported in SSA (58, 60, 68).

## Study strengths

As far as we know, this is the first study in SSA to assess the dietary intake, cardiometabolic profiles and other related NCD risk factors simultaneously among PLWH. The use of MSM to obtain usual intake instead of the average intake as seen in most of the previous studies is an added strength of this study. Moreover, for comparison of energy and nutrient intake, we used more individual suited DRIs (EAR, AI and AR) instead of RDA. RDA may overestimate the nutrient requirements for an individual since its best suited as population-wide recommendation for nutrient intake (29, 30). The use of POCT for lipid profile is an invaluable rapid NCD risk finding technique especially in resource-constrained settings (69).

## Study limitations

The 24-h dietary recall is prone to misreporting of the actual intake and food seasonality bias. As such the season of the study can have influence on the dietary intake of participants. Another potential drawback is that participants were recruited by a non-random sampling technique which introduces a sampling bias. Moreover, since participants were drawn from only Wakiso district which is composed of both urban and peri-urban settings, our results may not be representative of the general HIV community care model. There's still contention on dependability of total cholesterol POCT results (70). The use of IPAQ to assess physical activity has both intrinsic and extrinsic pitfalls such as recall bias and overestimation (71).

## Conclusion

Our findings reveal that metabolic disturbances exist at high levels among ART-treated patients in Uganda. This study further highlights the inadequate intake of fruits and vegetables underlining the need for dietary optimisation to improve both macro and micronutrient intake.

## Data availability statement

The original contributions presented in the study are included in the article/Supplementary material, further inquiries can be directed to the corresponding author.

## Ethics statement

The studies involving human participants were reviewed and approved by Uganda National Council for Science and Technology. The patients/participants provided their written informed consent to participate in this study.

## Author contributions

TK, CM, PO, BM, PY, and FK conceived and designed the study. TK and FK contributed to data collection and sample analysis. TK, PY, BV, and CM analyzed, interpreted the data, wrote, edited, and reviewed the manuscript. All authors read and approved the final version.

## Funding

This study was funded by the Belgian Directorate General for Development Cooperation and Humanitarian Aid (DGD), for funding through the VLIR-UOS framework.

## Conflict of interest

The authors declare that the research was conducted in the absence of any commercial or financial relationships that could be construed as a potential conflict of interest.

## Publisher's note

All claims expressed in this article are solely those of the authors and do not necessarily represent those of their affiliated organizations, or those of the publisher, the editors and the reviewers. Any product that may be evaluated in this article, or claim that may be made by its manufacturer, is not guaranteed or endorsed by the publisher.
