# Supplementary material for: The cardiometabolic profile and related dietary intake of Ugandans living with HIV and AIDS
Source: Front Nutr. 2022 Aug 11;9:976744. doi: 10.3389/fnut.2022.976744 (PMC9403861; doi:10.3389/fnut.2022.976744)
Supplement: Supplementary file 1 [file Table_1.DOCX]

Supplementary Material

**Supplementary Table 1: Proportions of metabolic syndrome and it’s components and high BMI classified by age**

| Age (years) | Metabolic syndrome | Elevated TG>150mg/dL | Elevated FBG>100mg/dL | WC  Male>94cm Female>80cm | Low HDL:  Male>40mg/dL Female>50mg/dL | High SBP>130mmHg | High DBP>85mmHg | BMI | |
| --- | --- | --- | --- | --- | --- | --- | --- | --- | --- |
|  | (n, %) | (n, %) | (n, %) | (n, %) | (n, %) | (n, %) | (n, %) | Overweight (24.9-29.9) (n, %) | Obesity (>30)  (n, %) |
| **20-29** |  |  |  |  |  |  |  |  |  |
| Total (n=29) | 4(13.8) | 4(13.8) | 8(27.6) | 9(31) | 17(58.6) | 7(24.1) | 2(6.9) | 7(28) | 5(20) |
| M (n=4) | 0(0) | 0(0) | 2(50) | 0(0) | 1(25) | 3(75) | 0(0) | 0(0) | 0(0) |
| F (n=25) | 4(16) | 4(16) | 6(24) | 9(36) | 16(64) | 4(16) | 2(8) | 7(31.8) | 5(22.7) |
| **30-39** |  |  |  |  |  |  |  |  |  |
| Total (n=80) | 26(32.5) | 17(21.2) | 36(45) | 25(31.2) | 49(61.2) | 21(26.2) | 4(5) | 22(28.9) | 21(27.6) |
| M (n=21) | 7(33.3) | 3(14.3) | 10(47.6) | 0(0) | 8(38.1) | 6(28.6) | 0(0) | 3(15.4) | 9(45) |
| F (n=59) | 19(32.2) | 14(23.7) | 26(44.1) | 25(42.4) | 41(69.5) | 15(25.4) | 4(6.8) | 19(33.9) | 12(21.4) |
| **40-49** |  |  |  |  |  |  |  |  |  |
| Total (n=83) | 25(30.1) | 22(26) | 46(55.4) | 44(53.0) | 42(50.6) | 25(30.1) | 6(7.2) | 28(37.8) | 18(24.3) |
| M (n=25) | 11(44) | 4(16) | 15(60) | 2(8.0) | 10(40.0) | 9(36) | 3(12) | 12(54.5) | 4(18.2) |
| F (n=58) | 14(24) | 18(31) | 31(53.4) | 42(72.4) | 32(55.2) | 16(27.6) | 3(5.2) | 16(30.8) | 14(26.9) |
| **>50** |  |  |  |  |  |  |  |  |  |
| Total (n=61) | 16(26.2) | 25(41) | 36(59) | 36(59.0) | 37(60.7) | 27(44.3) | 2(3.3) | 20(34.5) | 9(15.5) |
| M (n=23) | 7(30.4) | 6(26.1) | 13(56.5) | 7(30.4) | 8(34.8) | 8(34.8) | 0(0) | 12(57.1) | 3(14.3) |
| F (n=38) | 9(23.7) | 19(50) | 23(60.5) | 29(76.3) | 29(76.3) | 19(50.0) | 2(5.3) | 8(21.6) | 6(16.2) |

WC: Waist circumference, TG: Triglycerides, SBP: Systolic Blood Pressure, DBP: Diastolic Blood Pressure, HDL: High Density Lipoproteins

Supplementary Table 2: Energy (kcal) and Total dietary fibre distribution per eating occasion

| Meal | Total | | |  | Male | | |  | Female | | |
| --- | --- | --- | --- | --- | --- | --- | --- | --- | --- | --- | --- |
|  | Energy(kcal) *Mean (SD)* | Total dietary fibre (g) *Mean (SD)* | n |  | Energy(kcal) *Mean (SD)* | Total dietary fibre (g) *Mean (SD)* | n |  | Energy(kcal) *Mean (SD)* | Total dietary fibre (g) *Mean (SD)* | n |
| Breakfast | 752(459) | 5(8) | 233 |  | 727(415) | 5(7) | 66 |  | 761(476) | 6(8) | 167 |
| Midmorning snack | 223(292) | 2(7) | 35 |  | 143 (169) | 4(13) | 10 |  | 256(326) | 1(2) | 25 |
| Lunch | 969(516) | 12(14) | 244 |  | 1029(529) | 13(12) | 72 |  | 945(510) | 15(15) | 172 |
| Evening snack | 248(330) | 1.3(3) | 50 |  | 317(440) | 1(3) | 16 |  | 215(264) | 1(2) | 34 |
| Dinner | 755(551) | 9(10) | 233 |  | 749(306) | 8(7) | 69 |  | 758(602) | 10(10) | 164 |
| Night snack | 375(302) | 0.4(1) | 26 |  | 398(311) | 0(0) | 8 |  | 365(306) | 0.5(1) | 18 |

Supplementary Figure 1: Usual consumption of different food groups
